# Comparative Ubiquitination Proteomics Revealed the Salt Tolerance Mechanism in Sugar Beet Monomeric Additional Line M14

**DOI:** 10.3390/ijms232416088

**Published:** 2022-12-17

**Authors:** He Liu, Jialin Zhang, Jinna Li, Bing Yu, Sixue Chen, Chunquan Ma, Haiying Li

**Affiliations:** 1 Engineering Research Center of Agricultural Microbiology Technology, Ministry of Education & Heilongjiang Provincial Key Laboratory of Ecological Restoration and Resource Utilization for Cold Region & School of Life Sciences, Heilongjiang University, Harbin 150080, China; 2 Heilongjiang Provincial Key Laboratory of Plant Genetic Engineering and Biological Fermentation Engineering for Cold Region & Key Laboratory of Molecular Biology, College of Heilongjiang Province, Heilongjiang University, Harbin 150080, China; 3 Department of Biology, University of Mississippi, Oxford, MS 38677, USA

**Keywords:** ubiquitination, sugar beet M14, salt stress, label-free proteomics, molecular networks

## Abstract

Post-translational modifications (PTMs) are important molecular processes that regulate organismal responses to different stresses. Ubiquitination modification is not only involved in human health but also plays crucial roles in plant growth, development, and responses to environmental stresses. In this study, we investigated the ubiquitination proteome changes in the salt-tolerant sugar beet monomeric additional line M14 under salt stress treatments. Based on the expression of the key genes of the ubiquitination system and the ubiquitination-modified proteins before and after salt stress, 30 min of 200 mM NaCl treatment and 6 h of 400 mM NaCl treatment were selected as time points. Through label-free proteomics, 4711 and 3607 proteins were identified in plants treated with 200 mM NaCl and 400 mM NaCl, respectively. Among them, 611 and 380 proteins were ubiquitinated, with 1085 and 625 ubiquitination sites, in the two salt stress conditions, respectively. A quantitative analysis revealed that 70 ubiquitinated proteins increased and 47 ubiquitinated proteins decreased. At the total protein level, 42 were induced and 20 were repressed with 200 mM NaCl, while 28 were induced and 27 were repressed with 400 mM NaCl. Gene ontology, KEGG pathway, protein interaction, and PTM crosstalk analyses were performed using the differentially ubiquitinated proteins. The differentially ubiquitinated proteins were mainly involved in cellular transcription and translation processes, signal transduction, metabolic pathways, and the ubiquitin/26S proteasome pathway. The uncovered ubiquitinated proteins constitute an important resource of the plant stress ubiquitinome, and they provide a theoretical basis for the marker-based molecular breeding of crops for enhanced stress tolerance.

## 1. Introduction

Since the discovery of ubiquitin (Ub) in 1975, it has been found to play a critical and irreplaceable role in the growth, development, health, and diseases of living organisms [1]. Ubiquitination reactions usually require the synergistic actions of ubiquitin-activating enzymes (E1s), ubiquitin-conjugating enzymes (E2s), and ubiquitin-ligase enzymes (E3s) [2]. Different types of ubiquitination modifications generate unique downstream signaling processes [3,4,5]. Notably, ubiquitination not only can happen to any nascent or newly translated and misfolded protein but can also participate in the molecular regulatory processes by which organisms respond to various environmental factors. Therefore, exploring candidate resistant post-translational modifications (PTMs) with ubiquitination proteomics becomes a new strategy for gaining insight into the molecular mechanisms of organismal growth, development, and stress resilience.

Although ubiquitination modifications are a major type of PTM, they only account for 1% or less of the total proteome [6]. Even when both proteasomes and deubiquitinating enzymes are inhibited, the accumulation of ubiquitin-modified proteins remains limited in cells. Therefore, the most critical step in the high-throughput identification of ubiquitinated proteins by LC-MS/MS is the development of strategies to enrich ubiquitinated proteins. Currently, ubiquitin-coupled tagging, ubiquitin affinity media, and antibodies are the main methods used for the enrichment of ubiquitinated proteins. About two decades ago, ubiquitination sites of yeast (*Saccharomyces cerevisiae*) proteins were first identified on a large scale using mass spectrometry [7]. Subsequently, tandem ubiquitin binding domains (UBDs) have been used to specifically enrich ubiquitinated proteins [8]. In recent years, significant breakthroughs in peptide-targeted enrichment strategies have been made in the identification of ubiquitination sites [9]. The emergence of K-ε-GG antibodies has led to significant progress in large ubiquitination proteomic studies in rice (*Oryza sativa* L.), wheat (*Triticum aestivum* L.), and peach (*Prunus persica* L.) [10,11,12,13].

With the technological advancement in LC-MS/MS, the ubiquitination proteomics of plant signal transduction and stress responses have become a current research hotspot, such as light regulation, hormone signaling, and biotic/abiotic stresses. In *Arabidopsis thaliana*, an investigation of differentially ubiquitinated proteins revealed the mechanism of protein reassembly during red-light-induced photomorphogenesis in etiolated seedlings [14]. The ubiquitination proteomics of maize seedlings uncovered multiple ubiquitination sites in photosynthesis proteins and light-signaling proteins, implying their unique regulatory roles [15]. The overall level of protein ubiquitination in *Petunia* was increased in ethylene-mediated corolla senescence, suggesting an important role of protein ubiquitination modification [16]. In the tea plant (*Camellia sinensis* L), ubiquitination modifications are involved in plant resistance to drought stress [17]. Additionally, ubiquitinated proteins provide a valuable resource for understanding how the ubiquitination system regulates the rice immune response to pathogen infection [18]. Although several plant ubiquitination systems have been studied for their roles in plant responses to environmental factors, the ubiquitination proteomics of sugar beet, the world’s major sugar-producing crop, have not been reported. The sugar beet monomeric additional line M14 was obtained from interspecific crosses and backcrosses between cultivated beet (*Beta vulgaris* L.) and wild white flowering beet (*B. corolliflora* Zoss), which has a high salt tolerance [19]. In previous studies, sugar beet M14 was proven to exhibit salt tolerance, and its underlying molecular mechanisms have been explored through omics studies, e.g., membrane proteomics, phosphorylation proteomics, and redox proteomics [20,21,22,23,24]. The results highlight the importance of protein folding and degradation-related proteins in sugar beet salt tolerance [25].

There are functional interactions and crosstalk between the modifying effects of ubiquitination and phosphorylation in cellular signal transduction. For example, the phosphorylation modification of one or several sites on a substrate protein can induce the ubiquitination and subsequent degradation of the substrate [26]. Phosphorylation modifications can also disrupt the interaction between the E3 ubiquitin ligase and its substrates [27]. In addition to phosphorylation regulating ubiquitination, ubiquitination can also affect phosphorylation by regulating protein kinase activity [28]. Overall, ubiquitination primarily affects kinase signaling through proteosome degradation, while phosphorylation acts as a multifunctional switch in the ubiquitination pathway. Thus, ubiquitination proteomics and PTM crosstalk can be expected to provide an improved understanding of the molecular regulations underlying salt tolerance in sugar beet.

In this work, combining the results of qRT-PCR and Western blot assays, optimal time points for ubiquitination modification in the roots of the sugar beet monomeric additional line M14 in response to salt stress were determined. The differentially expressed ubiquitinated proteins (DEUPs) and ubiquitination sites were mapped using quantitative label-free proteomics. A bioinformatic analysis of the DEUPs was carried out to explore potential salt tolerance mechanisms of the sugar beet M14 line from the perspective of ubiquitination regulation and PTM crosstalk.

## 2. Results

### 2.1. Determination of the Response Time of Ubiquitination Modifications to Salt Stress

**Figure 1:**
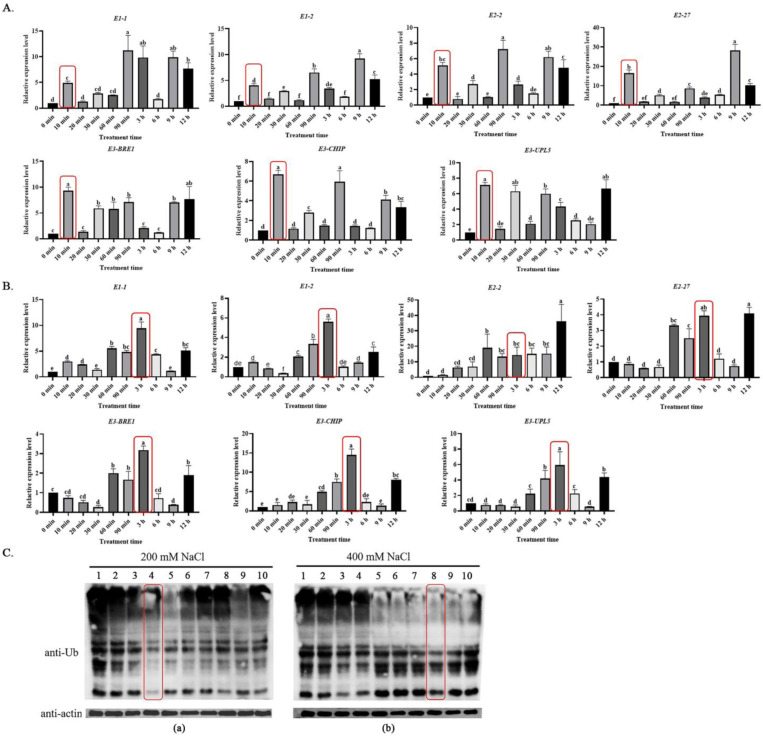
Gene transcription and protein level responses of the ubiquitin system in the roots of sugar beet M14 after salt stress treatments. (**A**) qRT-PCR of key genes in the ubiquitin system after 200 mM NaCl treatment. (**B**) qRT-PCR of key genes in the ubiquitin system after 400 mM NaCl treatment. The red-rectangle-enclosed bars represent the initial response time of key enzyme genes in the ubiquitin system to salt stress. Data were analyzed by Duncan’s analysis of variance, and different lowercase letters (a, b, c, d, e, f) indicate differences in genes expression. (**C**) Western blot analysis of protein ubiquitination levels after (**a**) 200 mM NaCl and (**b**) 400 mM NaCl treatments. The red-rectangle-enclosed lanes represent the point in time when the overall ubiquitination protein was significantly reduced. Lanes 1–10 represent the samples after different times of NaCl treatment, respectively: 1, 0 min; 2, 10 min; 3, 20 min; 4, 30 min; 5, 60 min; 6, 90 min; 7, 3 h; 8, 6 h; 9, 9 h; 10, 12 h. Different letters above bars denote statistical differences.

Ubiquitin modification occurs in organisms at any time, but the time dynamics for protein ubiquitination under biotic or abiotic stresses is important for exploring the response mechanisms. To investigate the response and regulatory mechanisms of protein ubiquitination modifications under plant salt stress, seven key enzyme genes (*BvM14-E1-1* (XP_010676802.1), *BvM14-E1-2* (XP_010685926.1), *BvM14-E2-1* (XP_010678313.1), *BvM14-E2-2* (XP_010683242.1), *BvM14-E3-1* (XP_010686720.1), *BvM14-E3-2* (XP_010692108.1), and *BvM14-E3-3* (XP_010666937.1)) of the sugar beet ubiquitination system were examined for changes in transcript levels. It was found that the expression of ubiquitination system genes increased rapidly at 10 min of low salt stress (200 mM NaCl) (Figure 1A), while the expression of genes increased significantly after 3 h of high salt stress (400 mM NaCl) (Figure 1B). This phenomenon was seemingly caused by the fact that sugar beet seedlings can be promoted to grow at low salt concentrations, while they are subjected to different degrees of damage under high salt stress, which triggers different molecular regulatory mechanisms [29]. In experiments measuring the ubiquitination levels of sugar beet proteins after salt stress, an interesting phenomenon triggered us to think about the pattern of protein ubiquitination modifications under salt stress. We found the ubiquitination level of total protein was reduced and the content of small molecules of ubiquitin decreased at 30 min (lane 4) of low-salt treatment, and the overall protein ubiquitination modifications in the plants remained at low levels. A significant decrease in ubiquitination modification was also observed at 6 h (lane 8) of high-salt treatment (Figure 1C). Combining the results of the qRT-PCR and Western blot assays, the optimal time points for ubiquitination changes in response to salt stress were determined to be 30 min for the 200 mM NaCl treatment and 6 h for the 400 mM NaCl treatment.

### 2.2. LC-MS/MS Identification of Ubiquitinated Proteins

**Figure 2:**
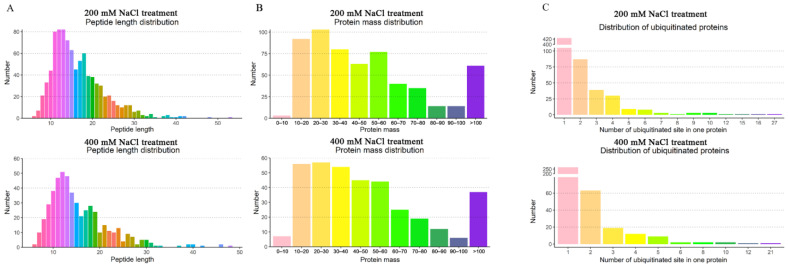
Distribution characterization of the identified ubiquitinated proteins. (**A**) Length distribution of the identified peptides derived from proteins in the roots of the sugar beet M14 line. (**B**) Mass distribution of the identified proteins in the sugar beet M14 line. (**C**) Distribution of the ubiquitination sites in the identified peptides derived from proteins in the sugar beet M14 roots. (**Top panel**), 200 mM NaCl treatment; (**Bottom panel**), 400 mM NaCl treatment.

In total, 4711 and 3607 proteins were identified in sugar beet roots after the 200 mM NaCl and 400 mM NaCl treatments, respectively. Among the identified proteins, 611 and 380 were ubiquitinated proteins with 1085 and 625 ubiquitination modification sites, respectively (Tables S1 and S2). In addition, the ubiquitinated peptide lengths ranged from 6 to 53 amino acids, with the majority being 10–30 amino acids (Figure 2A). The masses of the identified ubiquitinated proteins were in the range of 10–60 kDa, with the majority being 20–30 kDa (Figure 2B). Although most of the proteins had one ubiquitination site, fructose-bisphosphate aldolase 6, polyketide cyclase/dehydrase and lipid transport superfamily protein, actin-7, ATPase 2, cell division fructose-bisphosphate aldolase 6, biquitin-NEDD8-like protein RUB1, and histone H2B.6 had more than 10 ubiquitinated modification sites (Table S3). Ubiquitin-NEDD8-like protein RUB1 was identified in both the low- and high-concentration NaCl treatments. RUB1 was the protein with the most ubiquitination sites, with 21 and 27 sites identified under 200 mM NaCl and 400 mM NaCl, respectively. Ubiquitin-like proteins can regulate ubiquitination modifications and the cell cycle by activating RING-type E3 ubiquitin ligases. In addition to RUB1, there were a few proteins with more than 10 ubiquitination sites, whose functions need to be further explored and verified (Figure 2C). It is hypothesized that these ubiquitinated proteins may be tightly regulated by ubiquitination/deubiquitination to perform their functions under salt stress.

### 2.3. Functional Classification of the Differentially Expressed Ubiquitinated Proteins

**Figure 3:**
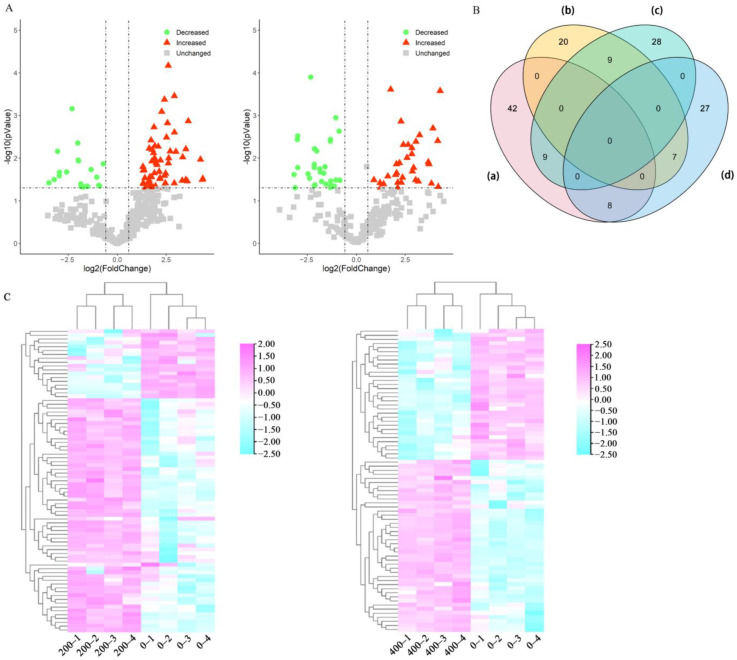
Quantitative analysis of the DEUPs. (**A**) Volcano plots of the DEUPs under 200 mM NaCl treatment (**left panel**) and 400 mM NaCl treatment (**right panel**). (**B**) Venn diagram of the DEUPs: (**a**) increased DEUPs under 200 mM NaCl treatment; (**b**) decreased DEUPs under 200 mM NaCl treatment; (**c**) increased DEUPs under 400 mM NaCl treatment; and (**d**) decreased DEUPs under 400 mM NaCl treatment. (**C**) Heat map of the hierarchical clustering of the DEUPs under 200 mM NaCl treatment (**left panel**) and 400 mM NaCl treatment (**right panel**).

In total, 70 DEUPs increased and 47 DEUPs decreased. Among them, 42 DEUPs increased and 20 DEUPs decreased under low salt stress, and 28 DEUPs increased and 27 DEUPs decreased under high salt stress (Tables S4 and S5, *p* < 0.05 and fold change >2 or <0.5). Many of the ubiquitinated proteins were found to change greatly before and after salt stress, indicating the activation of the cellular ubiquitination/deubiquitination system (Figure 3A,B). The results of the cluster analysis showed the good reproducibility of the samples in the control and treatment groups. Moreover, the differentially ubiquitinated proteins were well differentiated (Figure 3C). The ubiquitinated proteins may be degraded and/or reverted to non-ubiquitinated proteins by deubiquitination due to the presence of deubiquitinating enzymes. Therefore, some of the ubiquitinated proteins were present in either the control group or the treatment group. A total of 92 ubiquitin proteins were identified only in the control group, while 94 ubiquitin proteins were identified only in salt stress. Specifically, 64 ubiquitinated proteins were found only in the 200 mM NaCl-treated group, 30 ubiquitinated proteins were found in the 400 mM NaCl groups. Here, we found that isocitrate dehydrogenase and phosphoenolpyruvate carboxylase 3, two key enzymes in the tricarboxylic acid cycle, were ubiquitinated under different levels of salt stress. YchF (Obg-like ATPase 1) is a member of the Obg family, and the expression of YchF1 suppresses antioxidation enzymatic activities and increases lipid peroxidation in transgenic *Arabidopsis* and leads to the accumulation of reactive oxygen species (ROS) in tobacco [30]. In the present study, it was increased in ubiquitination and may be targeted to the 26S proteasome for degradation, preventing the excessive accumulation of ROS under salt stress and enhancing sugar beet salt tolerance. In addition, V-type proton ATPase subunit E3 was significantly increased in ubiquitination, suggesting that ubiquitination may also be involved in the H^+^ transport process, providing the impetus for intracellular ion transport and thus improving salt tolerance [31]. The phospholipid hydroperoxide glutathione peroxidase 6 was significantly decreased in ubiquitination, which may attenuate the cellular damage caused by lipid peroxidation, suggesting that ubiquitination may mitigate the cellular damage caused by salt stress. The aquaporin PIP1-4, important for water transport across membranes, can alter the cellular osmotic potential. Many aquaporins can improve salt tolerance in plants, but excessive water transport may have detrimental effects on plants [32,33]. PIP1-4 was significantly decreased in ubiquitination, suggesting that the degradation of aquaporin can reduce water transport in sugar beet and thus improve its salt tolerance (Table 1).

### 2.4. Functional Analyses of the Differentially Expressed Ubiquitinated Proteins

#### 2.4.1. Gene Ontology Enrichment

**Figure 4:**
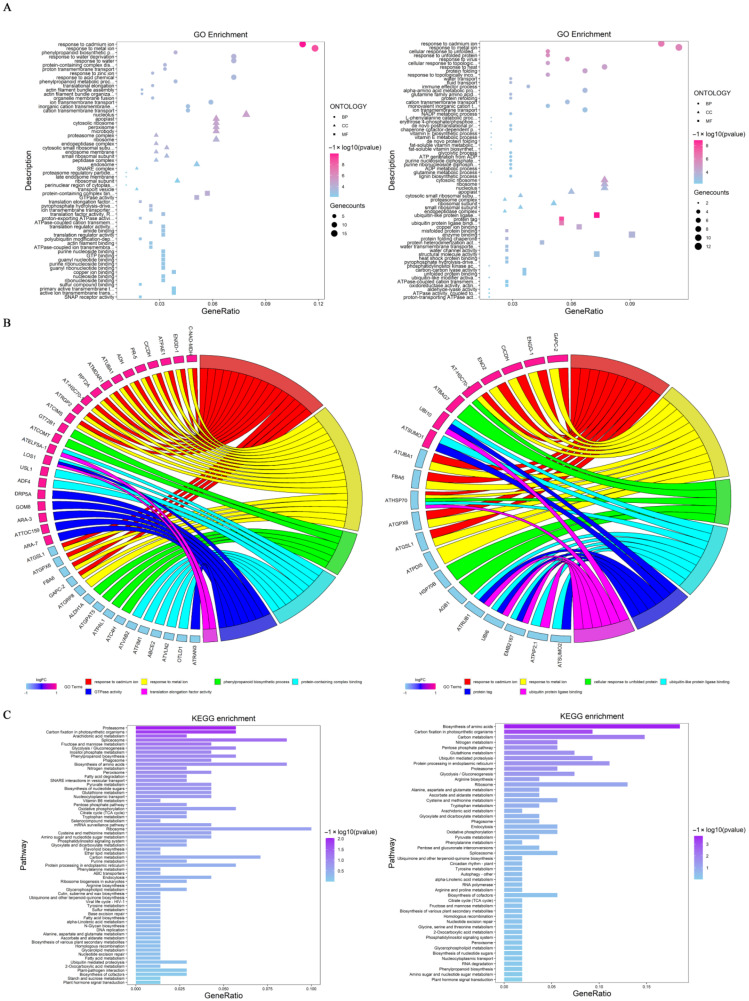
Bioinformatics analysis of the DEUPs. (**A**) GO enrichment bubble chart of the DEUPs ((**left panel**), 200 mM NaCl; (**right panel**), 400 mM NaCl). See Table S6 for specific notes. (**B**) Ubiquitinated protein distribution of main enrichment pathways (pink, increased ubiquitinated proteins; blue, decreased ubiquitinated proteins). (**C**) KEGG enrichment of the DEUPs ((**left panel**), 200 mM NaCl; (**right panel**), 400 mM NaCl). Refer to Table S7 for detailed information.

To unravel the potential molecular mechanisms of ubiquitination/deubiquitination in response to salt stress, a gene ontology (GO) functional enrichment analysis of DEUPs was performed in terms of biological processes (BPs), cellular components (CCs), and molecular functions (MFs). The results showed significant differences between the two treatments groups with different salt concentrations (Figure 4A). The main categories of BPs of the 200 mM NaCl group were responses to metal ions, the phenylpropanoid biosynthetic process, responses to water deprivation, and protein-containing complex disassembly. The 400 mM NaCl group were significantly enriched with responses to metal ions, unfolded proteins and viruses, and protein folding. For CC, the DEUPs were mainly related to the nucleolus, apoplast, cytosolic ribosome, peroxisome, and microbody in the 200 mM NaCl group, while the cytosolic ribosome, ribosomal subunits, and nucleolus were enriched in the 400 mM NaCl group. For MF, protein-containing complex binding and GTPase activity were the most enriched terms in the 200 mM NaCl group, while ubiquitin-like protein ligase binding, copper ion binding, and misfolded protein binding were shown in the 400 mM NaCl group. These results clearly showed that the ubiquitination/deubiquitination responses in the roots to the 200 mM and 400 mM NaCl treatments were different.

The relationship between the DEUPs and GO pathways was not a simple one-to-one correspondence but a complex one-to-many relationship (Figure 4B). Under 200 mM NaCl, alcohol dehydrogenase and plasma membrane ATPases were found to be necessary for root survival and adaptation to the stress. Most GO pathways were enriched for both increased and decreased DEUPs, e.g., the DEUPs enriched in GTPase activity, pyrophosphate-hydrolysis-driven proton transmembrane transporter activity, and ion transmembrane transporter activity were all increased in ubiquitination, which may play a positive regulatory role in the plant response to salt stress. Under 400 mM NaCl, heat shock 70 protein 1 was enriched in five different pathways and aquaporin PIP1-4 was concurrently enriched in seven pathways. This result suggests that these two proteins had multiple functions. In addition, the GO pathways of response to unfolded protein and misfolded protein binding were enriched, suggesting that ubiquitination was mainly involved in the degradation of misfolded proteins.

#### 2.4.2. KEGG Pathway Enrichment

In order to gain insight into the metabolic pathways that the DEUPs are involved in, a KEGG pathway enrichment was performed (Figure 4C). Under 200 mM NaCl, most of the DEUPs were involved in the proteasome, carbon fixation in photosynthetic organisms, arachidonic acid metabolism, the spliceosome, and the biosynthesis of amino acids. Among them, four proteins (P42742, Q9SSB5, O81149, and Q9SZD4) were enriched in the proteasome pathway, all of which were involved in protein degradation of the proteasome components, suggesting turnover of the proteosome components in response to low salt stress. Under 400 mM NaCl, most DEUPs were enriched in the biosynthesis of amino acids, carbon fixation in photosynthetic organisms, carbon metabolism, nitrogen metabolism, and the pentose phosphate pathway. The result was consistent with previous observations of decreased biosynthesis and growth under high salt stress [22,34].

### 2.5. Protein–Protein Interactions of Differentially Ubiquitinated Proteins

**Figure 5:**
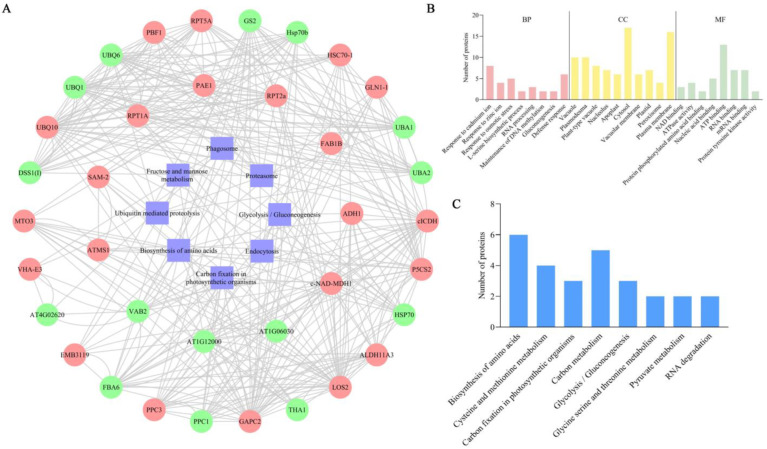
Interacting protein map of the DEUPs and phosphorylation site analysis of key DEUPs. (**A**) Predicted map of the DEUP interactions. (**B**) GO functional annotation of ubiquitinated and phosphorylated co-modified proteins. (**C**) KEGG functional annotation of the ubiquitinated and phosphorylated co-modified proteins.

To further understand the potential interactions between DEUPs, interaction predictions were made for eight significantly enriched KEGG pathways (proteasome, carbon fixation in photosynthetic organisms, fructose and mannose metabolism, glycolysis/gluconeogenesis, phagosome, biosynthesis of amino acids, endocytosis, and ubiquitin-mediated proteolysis). The outer circle shows the DEUPs under the 200 mM NaCl treatment, and the inner circle shows the DEUPs under the 400 mM NaCl treatment (Figure 5A). The protein interactions were found to be very complex, and the proteins were closely linked to each other.

### 2.6. Crosstalk between Phosphorylation and Ubiquitination

A total of 76 proteins were identified as having both ubiquitination and phosphorylation modifications (Table S8). The possible crosstalk between ubiquitination and phosphorylation on these proteins provides new insight into the molecular regulatory mechanisms underlying the sugar beet M14 tolerance to salt stress. As shown in Figure 5B, the largest group of proteins was involved in the response to cadmium ions and the defense response processes, and in terms of molecular functions the largest group of proteins was involved in ATP binding, RNA binding, and mRNA binding. The results suggest that ubiquitination and phosphorylation co-modified proteins may respond to salt stress through regulating cellular transcriptional, energy, and stress/defense-related processes.

A KEGG annotation of the co-modified proteins was performed, and the results are shown in Figure 5C. Under salt stress conditions, the co-modified proteins were involved in amino acid biosynthesis; cysteine and methionine metabolism; carbon fixation in light and biology; carbon metabolism; glycolysis/gluconeogenesis; glycine, serine, and threonine metabolism; pyruvate metabolism; and RNA degradation. The results indicate that proteins related to amino acid synthesis as well as carbon-metabolism-related pathways are susceptible to coregulation by ubiquitination and phosphorylation modifications under salt stress.

Additionally, a number of transport- and metabolism-related proteins were identified as being modified by both ubiquitination and phosphorylation, presumably as ubiquitinated substrate proteins that are first phosphorylated and subsequently recognized and ubiquitinated by the corresponding E3 ubiquitin ligases, thereby regulating their function or abundance in response to salt stress. Moreover, two E3 ubiquitin ligases (Q0WPJ7 and Q94AH6) were identified that underwent both phosphorylation and ubiquitination. It is possible that their activities may be regulated by certain protein kinases. Three protein kinases (B9DFG5, Q9M0X5, and O65468) were also identified, highlighting their regulation by ubiquitination, which allows the precise control of intracellular signaling pathways.

## 3. Discussion

**Figure 6:**
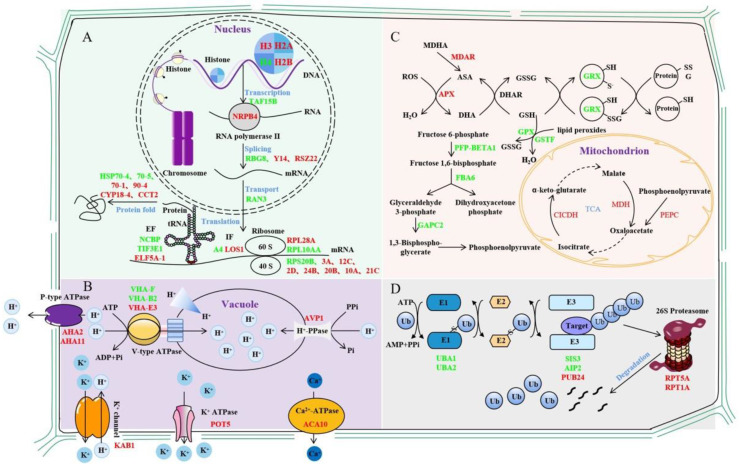
Mechanisms of ubiquitinated protein changes in sugar beet roots under different concentrations of salt stress. (**A**). Mechanism of transcriptional and translational regulation of ubiquitination in sugar beet M14 line in response salt stress. (**B**). Mechanism of signal transduction regulation of ubiquitination in sugar beet M14 line in response salt stress. (**C**). Mechanism of metabolism pathway regulation of ubiquitination in sugar beet M14 line in response salt stress. (**D**). Mechanism of UPS regulation of ubiquitination in sugar beet M14 line in response salt stress. Red represents increased ubiquitinated proteins, and green represents decreased ubiquitinated proteins.

With the advancement of proteomics technologies, researchers have gradually realized the importance of PTM mechanisms in plant stress responses and tolerance. In total, 70 and 47 DEUPs as well as 92 and 94 ubiquitinated proteins present only in the control and salt-treated groups were identified using a K-ε-GG antibody enriched with ubiquitin peptides for label-free quantitative proteomics. A complex molecular mechanism including transcription–translation, signal transduction, metabolism, and ubiquitin/26S proteasome pathways was proposed to highlight the first comprehension of ubiquitination functions in the sugar beet M14 salt stress response (Figure 6).

### 3.1. Ubiquitination Affects Gene Transcription and Translation Processes

Studies have shown that histone ubiquitination plays an important role in regulating many processes in the nucleus, especially in DNA damage repair [35]. For example, the RING-type E3 ubiquitin ligase RNF8 promotes DNA damage repair by ubiquitinating histone H2A/H2AX and thereby controlling the concentrations of repair proteins, e.g., Rap80, BRCA1, and 53BP1, at the site of DNA damage [36]. In our study, four histones were ubiquitinated, and the ubiquitinated histone H4 decreased, while the ubiquitinated histones H3, H2A, and H2B increased. Monoubiquitination often acts as a tag for substrate proteins to be targeted to a proteosome for degradation, and the monoubiquitination of histones H2A and H2B is a classic example of this process [37,38]. Here, several histone ubiquitin modifications were identified at altered levels, which may regulate the concentrations of repair proteins for promoting the repair of damaged DNA. In addition, the ubiquitination of the K1268 site of RPB1, the largest catalytic subunit of RNA polymerase II, enables its degradation, which is essential for the DNA damage repair process [39]. DNA-directed RNA polymerase II subunit 4, the second largest subunit of RNA polymerase II, was identified [40]. It synthesizes mRNA precursors and is a central component of the RNA polymerase II transcription machinery. Moreover, three transcription factors and five RNA-binding proteins were identified as being extensively involved in a variety of transcriptional RNA-binding events [41,42,43,44,45]. Cold shock protein 1, similar to glycine-rich RNA-binding protein 8, has RNAase activity and has been reported to be downregulated during salt stress [46,47]. The results of the present study suggest that this protein is ubiquitinated, which may lead to its degradation, thereby affecting its regulatory role on target genes.

The ubiquitination of translation initiation factors, translation elongation factors, and ribosomal proteins changed significantly after salt stress. For the translation initiation factors and translation elongation factors, the pattern of ubiquitination changes was not clear under the salt stress conditions. Notably, eight of the eleven identified ribosomal subunits were increased in ubiquitination. The result was consistent with previous findings of the presence of numerous ubiquitination modifications in various ribosomal subunits [11,48,49,50]. Studies in yeast have shown that ribosomal ubiquitination can be adapted to changing environmental conditions through selective deubiquitination mediated by the deubiquitinating enzyme Ubp3p/Bre5p [51]. The increased level of ubiquitination of ribosomal subunits under salt stress may promote ribosomal protein degradation and thus decrease protein synthesis [22,34]. In addition, heat shock proteins (HSPs) can promote protein folding to protect and maintain cell viability against adverse effects such as osmotic stress [52]. We found that HSP70-1 and HSP90-4 ubiquitination increased after low-salt treatment, while HSP70-5 and HSP70-4 ubiquitination decreased after high-salt treatment. Changes in the degree of ubiquitination of HSPs affect the stability of key proteins under salt stress conditions.

### 3.2. Ubiquitination Regulates Membrane Transport Processes

Three V-type ATPases (P0CAN7, Q9SZN1, and Q9ZQX4), one H^+^-PPase (P31414), and two P-type ATPases (P19456 and Q9LV11) were identified as having significantly altered levels of ubiquitin. V-type ATPases can acidify vesicles to provide energy for ion transport and metabolites [53]. V-type ATPase consists of two structural domains: a peripheral V1 domain consisting of eight different subunits (A-H) and an internal V0 domain with five different subunits (a, b, c, c’, and c”) [54]. Skp-Cul1-F-box (SCF) ubiquitin ligase promotes V-type ATPase subunit assembly and increases its activity [55]. The E3 ubiquitin ligase ZNRF2 can interact with V-type ATPase and affect its key function of maintaining organelle pH [56]. All three identified subunits of the V-type ATPase are located in its V1 domain and catalyze the hydrolysis of ATP while transporting protons into the lumens of vacuoles [57]. H^+^-PPase is a single-subunit protein that uses pyrophosphate (PPi) instead of ATP to produce a proton gradient within the vacuole membrane, leading to increased solute accumulation and water retention in the vacuole, thereby increasing salt tolerance in plants [58,59]. The P-type ATPase consumes ATP to transport H^+^ extracellularly, and the resulting external acidification or internal alkalinization of electrochemical gradients can be used by secondary transporters to facilitate the passage of ions and organic compounds across the plasma membrane [60,61,62]. The proton pump can transfer NaCl accumulated in the cell either by translocating Na^+^ into the vesicle (activated by V-type ATPase and H^+^-PPase) or expelling Na^+^ (activated by P-type ATPase) [63,64]. *PMA1p* encodes a P-type ATPase that alters intracellular pH when overexpressed. The E3 ubiquitin ligase Rsp5p can maintain intracellular pH homeostasis by ubiquitinating PMA1p to trigger endocytosis and degradation [65,66]. The ubiquitination levels of all types of protons were affected by salt stress, and the ubiquitin modification may promote the endocytosis of proton pumps to maintain intracellular pH homeostasis and prevent cellular damage.

Five secondary transporter proteins activated by proton pumps were identified, including aquaporins (Q39196, P43287, and P43286), Ca^2+^-ATPase (Q9SZR1), potassium transporter protein (Q9M7K4), and the potassium channel protein β subunit (O23016). Aquaporins are channel proteins that are required to facilitate water transport across membranes and have an important role in altering the cellular osmotic potential in response to salt stress [67,68,69]. The overexpression of PIP1b increases the sensitivity of tobacco to drought stress [70]. However, the RING-type E3 ubiquitin ligase Rma1H1 can ubiquitinate PIP2-1 and degrade it via the 26S proteasome, thereby reducing water transport and improving plant drought tolerance [71]. Notably, the aquaporin PIP2-1 (P43286) was identified, and both the level of ubiquitin modification and the protein level were significantly decreased under salt stress. It is speculated that this ubiquitination may act similarly to reduce water transport in sugar beet M14, thereby improving plant salt tolerance.

The ubiquitination levels of Ca^2+^-ATPase 10 (Q9SZR1), potassium transport protein 5 (Q9M7K4), and integrin-linked protein kinase 1 (F4IS56) were all identified as significantly increased under salt stress. Ca^2+^-ATPase (calcium-transporting ATPase) is a major Ca^2+^ efflux protein that transports Ca^2+^ from the cytoplasm against a concentration gradient to the plastid or pumps it into calcium-storing organelles such as the endoplasmic reticulum [72]. K^+^ is the most abundant cationic component in plants and has important functions in metabolism, growth, and stress adaptation [73]. The potassium transporter has been shown to be required to maintain plant growth at low K^+^ concentrations in the presence of salts [74]. In addition, potassium transporter proteins also mediate sodium transport and are involved in the plant response to salt stress [75]. Integrin-linked protein kinase interacts with potassium transport proteins and promotes potassium transport protein accumulation, thereby maintaining K^+^ homeostasis and improving salt tolerance in plants [60]. The RING-type E3 ubiquitin ligase CPN1 could ubiquitinate the potassium transport growth defect 1 (SKD1) without causing its degradation but only by altering its activity to maintain the dynamic Na^+^/K^+^ balance under salt stress [76]. It is possible that the ubiquitination signal does not directly lead to the degradation of the target proteins but rather maintains intracellular Ca^2+^ and K^+^ homeostasis in response to salt stress.

### 3.3. Ubiquitination Regulates Metabolic Pathways

This study identified a large number of ubiquitinated enzymes associated with different metabolic pathways. Carbohydrate metabolism provides plants with energy to cope with salt stress through glycolysis and the tricarboxylic acid cycle (TCA cycle) [77]. The key enzymes in the glycolytic pathway may have functions other than their own catalytic activity through PTMs [78]. For example, the E3 ubiquitin ligase SINAL7 changes its localization to the nucleus by monoubiquitinating 3-phosphoglyceraldehyde dehydrogenase, and this ubiquitination may confer additional functions to the protein in signal transduction [79]. Phosphoenolpyruvate carboxylase (PEPC) is a cytoplasmic enzyme that plays an important role in the non-photosynthetic and photosynthetic tissues of plants. The monoubiquitination of PEPC triggered selective autophagy of the enzyme to prevent cellular dysfunction [80,81]. In our work, the ubiquitin modification of three proteins involved in the TCA cycle were identified as significantly altered before and after salt stress, namely PEPC3 (Q84VW9), cytosolic isocitrate dehydrogenase (Q9SRZ6), and malate dehydrogenase 1 (P93819). It is hypothesized that the identified PEPC3 may undergo monoubiquitination to maintain normal cellular function, whereas isocitrate dehydrogenase may undergo both ubiquitination and SUMO modifications to maintain mitochondrial redox homeostasis under salt stress. SUMOylation is a ubiquitin-like modification mediated by a small ubiquitin-like modifier [82], and it enhanced the activity of isocitrate dehydrogenase [83]. The results suggest that the ubiquitination and/or SUMOylation of such enzymes involved in glycolysis and the TCA cycle may not only regulate the metabolic pathway to produce energy but may also regulate cellular stability in response to salt stress by altering enzyme functions.

The expression of the Obg family YchF (Obg-like ATPase 1) inhibits antioxidant enzyme activity and increases lipid peroxidation in transgenic Arabidopsis, leading to the accumulation of ROS [30,84,85]. The ubiquitination level of YchF (Q9SA73) was significantly increased and the protein level was significantly decreased under salt stress, suggesting ubiquitination-mediated degradation in the 26S proteasome, thereby preventing the excessive accumulation of ROS. Monodehydroascorbate reductase (MDAR) and ascorbate peroxidase (APX) are the key enzymes that catalyze the processes of ascorbate (AsA) production and H_2_O_2_ scavenging [86]. Glutathione peroxidase (GPX), glutathione S-transferase (GST), and aldehyde dehydrogenase (ALDH) can all mitigate the damage caused by lipid peroxidation [87]. Although their functions in ROS scavenging are well understood, studies related to ubiquitin modifications have not been reported. In the present study, we identified that the ubiquitination levels of MDAR (Q9LFA3), APX (Q42564), and GST (Q96266) increased and those of GPX (O48646) and ALDH (Q56YU0) significantly decreased in response to salt stress. The function of ubiquitination in association with the AsA-GSH cycle requires further study.

The overexpression of *OsGRX-C7* was shown to reduce the degree of membrane lipid peroxidation under salt stress through promoting the accumulation of proline and soluble sugar content and to improve the tolerance of rice to salt stress [88]. The bZIP (basic leucine zippers) transcription factor in plants can regulate a variety of processes, including development and the stress response [89]. *Os*bZIP47 in rice inhibits cell proliferation and has a negative effect on grain seed size. The E3 ubiquitin ligase GW2 can ubiquitinate a repressor of *Os*bZIP47 WG1, leading to its degradation, thereby weakening its repressive effect on *Os*bZIP47 and thus limiting cell growth [90]. In this experiment, the ubiquitination level of glutaredoxin (Q9FNE2) significantly decreased to maintain its protein abundance, and it may interact with other proteins to regulate the response of sugar beet M14 to salt stress.

### 3.4. Ubiquitination of UPS-Related Proteins under Salt Stress

Many important components of the ubiquitin-26S proteasome (UPS) pathway were DEUPs. Ubiquitin-activating enzyme E1 (UBA) is a negative regulator in response to salt stress in wheat, and the deletion of the UBA domain or mutation of the zinc finger domain can alleviate its negative regulatory effect and enhance salt tolerance in transgenic *Arabidopsis* [91]. Two UBA E1s (P93028 and P92974) showed decreased levels of ubiquitination under salt stress. Whether UBA also plays a negative role in the salt stress response of sugar beet M14 needs to be explored in further experiments.

E3 ubiquitin ligases are a central component of the UPS system. They recognize ubiquitinated substrates to control the specificity of ubiquitination [92]. In this work, two RING-type E3 ubiquitin ligases and one U-box-type E3 ubiquitin ligase were identified that showed significant changes in ubiquitination levels under salt stress. For example, the RING-H2 type E3 ubiquitin ligase SIS3 (Q8GYT9) had increased ubiquitination after salt stress. It may play an important role in the sugar response through the ubiquitin-26S proteasome pathway and in plant growth, development, and metabolism [93]. The U-box E3 ubiquitin ligase PUB24 (Q9SF15) has been reported to be associated with pathogen-associated molecular pattern (PAMP)-triggered immune (PTI) responses [94]. However, the functions of these ubiquitin ligases in salt stress have not been reported [93]. Abscisic acid (ABA)-insensitive 3 (ABI3) has been shown to regulate plant growth and developmental processes through ABA [95]. The RING-H2-type E3 ubiquitin ligase AIP2 (Q8RXD3) regulates protein hydrolysis and negatively regulates ABA signaling by targeting ABI3 polyubiquitination for 26S proteasome degradation. In summary, significant changes in the ubiquitination levels of three E3 ubiquitin ligases in the sugar beet M14 response to salt stress are new findings. Their functions in the salt stress response through regulating ABA or related pathways deserve further investigation.

Proteins modified by ubiquitination are recognized by various ubiquitin receptors and targeted to the 26S proteasome for degradation [96]. The ubiquitin receptors RAD (Q84L30) and DSK (Q9SII8) were both increased after salt stress, demonstrating that salt stress may activate the UPS system, leading to the degradation of a large number of ubiquitinated proteins [97,98]. The 26S proteasomes consist of two complexes, the 20S proteasome and the 19S regulatory complex. The regulatory complex consists of two parts: a basal complex and a cap complex. The six ATPase subunits in the basal complex, named RPT1-6, respectively, are responsible for the opening of the degradation channel and the substrate unfolding to help substrates enter the degradation lumen [99,100]. In our research, the ubiquitination levels of two substrate complex ATPase subunits, RPT1 (Q9SSB5) and RPT5 (Q9SEI2), were increased under salt stress, further demonstrating that the UPS system may be activated in response to salt stress. In addition, the ubiquitination modification levels of some deubiquitinating enzymes and some ubiquitin elongation proteins were significantly altered under salt stress, indicating that the ubiquitin system is very important in the response of plants to salt stress.

In this study, we explored the salt tolerance mechanism of sugar beet M14 from the perspective of ubiquitination regulation. In the future research, we will proceed from the following points: First, although ubiquitinated proteomics has yielded abundant data on ubiquitinated proteins in sugar beet M14, the specific functions of each ubiquitinated protein need to be verified by subsequent experiments on single proteins. Second, only two post-translational modifications, ubiquitination and phosphorylation, were integrated and analyzed, while the interactions between multiple PTMs were difficult to identify. In future experiments, the sequential enrichment of protein PTMs can effectively solve this problem. In the next stage of experiments, we will integrate the proteomic data of multiple PTMs to provide valuable genetic resources for subsequent experiments and to gain a more comprehensive and in-depth understanding of the dynamic network of PTMs to further reveal the molecular mechanism of salt tolerance in sugar beet M14.

## 4. Materials and Methods

### 4.1. Plant Material and Salt Treatment

The sugar beet monomeric additional line M14 was cultivated in a 1/2 concentration of Hoagland’s solution [101] with 13 h of light/11 h of darkness, 25 °C/20 °C diurnal temperatures, 450 μmol m^−2^s^−1^ light intensity, and 70% relative humidity. Five-week-old seedlings were used as experimental materials. The roots were collected, immediately frozen in liquid nitrogen, and stored at −80 ℃ after being stressed with 200 mM NaCl or 400 mM NaCl for 0 min, 10 min, 20 min, 30 min, 60 min, 90 min, 3 h, 6 h, 9 h, or 12 h.

### 4.2. Quantitative Real-Time PCR (qRT-PCR)

Total RNA was extracted from 5-week-old sugar beet roots after the NaCl treatments using Trizol (Sangon Biotech, Shanghai, China). cDNA synthesis was performed with a PrimeScript™ RT Master Mix kit (Takara, Dalian, China). Primers were designed using primer 3 plus (www.primer3plus.com (accessed on 15 November 2022) and NCBI-primer blast (www.ncbi.nlm.nih.gov/tools/primer-blast (accessed on 15 November 2022) online websites (Table S9). SYBR™ Green I dye (Applied Biosystems™, Foster City, CA, USA) was used for qRT-PCR.

### 4.3. Protein Extraction and Western Blot

Roots were ground to powder in liquid nitrogen and suspended in a 30 mL tube containing 5 mL of Tris-saturated phenol (TSP) (pH 8.8) and 5 mL of phenol extraction buffer (PEB) (25 mM TEAB, 10 mM EDTA, 200 mM DTT, 200 mM PMSF, and 900 mM sucrose) and centrifuged at 10,000× *g* at 15 °C for 10 min. The aqueous phase was added to 3 mL of TSP and 3 mL of PEB and centrifuged at 10,000× *g* at 15 °C for 10 min. The proteins were precipitated by adding five times the volume of 0.1 M ammonium acetate methanol (AAM) and standing overnight at −20 °C. The precipitate was washed twice with 10 mL of 0.1 M AAM, twice with 80% cold acetone, and once with 100% cold acetone. The acetone was removed by freeze drying, and the precipitate was dissolved in 1 mL of urea buffer (8 M urea, 0.5% SDS, 25 mM TEAB, and 1 mL of Triton X-100). Protein concentrations were measured and quantified using the Bradford Protein Assay Kit (TaKaRa, Dalian, China). Total proteins were electrophoresed with 12% SDS-PAGE and transferred to an Amersham Hybond P 0.45 PVDF membrane (Cytiva, 10600023, Marlborough, MA, USA). A ubiquitin (P37) antibody (Cell Signaling Technology (58395), Beverly, MA, USA) and a β-actin (D6A8) rabbit mAb (Cell Signaling Technology (8457), Beverly, MA, USA) were used in the immunoblotting analyses per the manufacturer’s instructions.

### 4.4. Trypsin Digestion and Desalination

Protein samples were reduced with 10 mM TCEP at room temperature for 1 h. After the digestion, peptides were dried and reconstituted in 0.1% formic acid in ultrapure water. The samples were desalted using solid-phase extraction (SPE) (The Nest Group, Sterling, USA) and Ziptip (Millipore, MMAS, Burlington, VT, USA) desalting columns following the manufacturers’ instructions.

### 4.5. Ubiquitinated Peptide Enrichment

The tryptic peptides were immunoaffinity-purified using a PTMScan^®^ Motif antibody conjugated to protein A agarose beads (Cell Signaling Technology (kit 5562), Beverly, MA, USA). Unbound peptides are removed by washing with IAP buffer, and the captured peptides containing the ubiquitin remnant motif (K-ε-GG) were eluted with 0.15% trifluoroacetic acid. The peptides are desalted with Ziptip and lyophilized for LC-MS/MS analysis.

### 4.6. LC-MS/MS

The samples were dissolved in reversed-phase LC solvent A (0.1% formic acid) and loaded onto an Easy-nLC-Orbitrap MS/MS system. The experiment was performed on an Easy-nLC1000 system (Thermo Fisher Scientific, Bremen, Germany) connected to a hybrid quadrupole Orbitrap mass spectrometer. The peptides were loaded onto an Acclaim C18 PepMap 100 pre-column (20 mm × 75 μm; 3 μm) and separated on a C18 PepMap RSLC analytical column (500 mm × 75 μm; 2 μm) at a flow rate of 300 nL/min over 110 min from solvent A to 30% solvent B (0.1% formic acid and 99.9% acetonitrile, *v*/*v*) in a linear gradient to 98% solvent B for a further 7 min. Full MS scans were obtained using a Q-Exactive Orbitrap mass analyzer in the *m*/*z* 400–2000 range with a resolution of 70,000 at 200 *m*/*z*. The 10 strongest peaks with more than two charges were separated by 1.3 *m*/*z* and fragmented in a high-energy collision cell using 28% normalized collision energy. The maximum ion injection time for the full spectrum scan and the MS/MS scan was 250 ms with ion target values set to 3e6 for full MS and 1e6 for MS/MS. The selected sequencing ions were dynamically excluded for 60 s.

### 4.7. Statistical Analysis

For qRT-PCR experiments, each reaction consisted of three biological replicates and three technical replicates. The relative expression levels of the target genes were calculated by normalizing to the 18S internal reference gene with ΔΔCt [102]. MS/MS profiles were searched using Proteome Discoverer 2.5 (Thermo Fisher Scientific, Bremen, Germany), searching the combined Sugar Beet Protein Database and the Green Plant Protein Database from NCBI (with a total of 6,255,663 entries). Trypsin was designated as the hydrolytic enzyme, allowing for two missing cleavages. The fragment mass error was 0.02 Da, and the peptide mass error was 10 ppm. At least three of the five biological replicates were quantified, and proteins with *p*-values < 0.05 and fold changes <0.5 or >2 were identified as differentially expressed proteins. The functional annotation of all proteins was performed by UniProt (http://www.ebi.uniprot.org (accessed on 15 November 2022). A KEGG pathway analysis of differentially expressed ubiquitinated proteins (DEUPs) was performed using the Kyoto Encyclopedia of Genes and Genomes (KEGG) (https://www.kegg.jp (accessed on 15 November 2022) website. GO annotation was performed via the online website AgriGO (http://bioinfo.cau.edu.cn/agriGO (accessed on 15 November 2022). Images were plotted through R language packages such as ggplot and GOplot. Heat maps were plotted and a clustering analysis was performed using the Heatpot module in TBtools. Protein interactions were displayed in Cytoscape.

## Figures and Tables

**Table 1:** Up/downregulated expression of ubiquitinated proteins in both the 200 mM and 400 mM NaCl treatment groups.

Accession ^a^	Uniport ^b^	Protein Name	Expression Change ^c^
731321402	Q9FK25	Flavone 3′-O-methyltransferase 1	Up
731337691	O65639	Cold shock protein 1	Up
731318867	Q9SRZ6	Cytosolic isocitrate dehydrogenase	Up
731349044	P22953	Heat shock 70 kDa protein 1	Up
731329055	Q84VW9	Phosphoenolpyruvate carboxylase 3	Up
731323478	Q940P8	T-complex protein 1 subunit beta	Up
731351524	Q9SA73	Obg-like ATPase 1	Up
731311373	Q9M156	UDP-glycosyltransferase 72B1	Up
731330860	P0CAN7	V-type proton ATPase subunit E3	Up
1108806148	O48646	Probable phospholipid hydroperoxide Glutathione peroxidase 6	Down
731311844	P53492	Actin-7	Down
733215449	Q39196	Probable aquaporin PIP1-4	Down
731345323	Q9SJQ9	Fructose-bisphosphate aldolase 6	Down
731372394	Q43127	Glutamine synthetase	Down
731328639	P59232	Ubiquitin-40S ribosomal protein S27a-2	Down
731368198	Q9SHE7	Ubiquitin-NEDD8-like protein RUB1	Down

^a^ Protein sequence number in the NCBI database. ^b^ Uniport protein number. ^c^ Changes in the abundance of ubiquitinated proteins in the 200 mM and 400 mM NaCl treatment groups.

## Supplementary Materials

The following supporting information can be downloaded at: https://www.mdpi.com/article/10.3390/ijms232416088/s1, Table S1. List of qRT-PCR primer sequences required for the assay. Table S2. DEUPs in the roots of sugar beet M14 plants under 200 mM NaCl stress. Table S3. DEUPs in the roots of sugar beet M14 plants under 400 mM NaCl stress. Table S4. Proteins with more than 10 ubiquitination sites in the sugar beet roots after NaCl treatments. Table S5. Proteins modified with both phosphorylation and ubiquitination in the sugar beet roots after NaCl treatments. Table S6 Specific content of GO enrichment. Table S7 Specific content of KEGG enrichment. Table S8 Sugar beet M14 line root proteins with both phosphorylations and ubiquitination. Table S9 Primer sequences required for the assay.
