# Supplementary material for: Comparative Ubiquitination Proteomics Revealed the Salt Tolerance Mechanism in Sugar Beet Monomeric Additional Line M14
Source: Int J Mol Sci. 2022 Dec 17;23(24):16088. doi: 10.3390/ijms232416088 (PMC9782053; doi:10.3390/ijms232416088)
Supplement: Supplementary file 1 [file ijms-23-16088-s001.zip › Table S3-Proteins with more than 10 ubiquitination sites in NaCl treatment.pdf]

Supplementary Table S3 Proteins with more than 10 ubiquitination sites in NaCl treatment

Accession[a]	Uniport[b]	Protein name[c]	Ubiquitinated site number with 200 mM NaCl	Ubiquitinated site number with 400 mM NaCl
731345323	Q9SJQ9	Fructose-bisphosphate aldolase 6	10	10
731351891	F4K5S5	Polyketide cyclase/dehydrase and lipid transport superfamily protein	10	-
731311848	P53492	Actin-7	12	10
731354199	P19456	ATPase 2	15	-
731351877	Q9LZF6	Cell division control protein 48 homolog E	16	-
731368198	Q9SHE7	Ubiquitin-NEDD8-like protein RUB1	27	27
731349098	O23629	Histone H2B.6	-	12

[a] Protein sequence number in the NCBI database. [b] Uniport protein number.
