# Supplementary material for: Comparative Ubiquitination Proteomics Revealed the Salt Tolerance Mechanism in Sugar Beet Monomeric Additional Line M14
Source: Int J Mol Sci. 2022 Dec 17;23(24):16088. doi: 10.3390/ijms232416088 (PMC9782053; doi:10.3390/ijms232416088)
Supplement: Supplementary file 1 [file ijms-23-16088-s001.zip › Table S6-List of GO enrichment entries.pdf]

Supplementary Table S6 List of GO enrichment entries

200 mM NaCl

1	response to cadmium ion
2	response to metal ion
3	phenylpropanoid biosynthetic process
4	response to water deprivation
5	response to water
6	protein-containing complex disassembly
7	proton transmembrane transport
8	response to zinc ion
9	response to acid chemical
10	phenylpropanoid metabolic process
11	translational elongation
12	actin filament bundle assembly
13	actin filament bundle organization
14	organelle membrane fusion
15	ion transmembrane transport
16	inorganic cation transmembrane transport
17	cation transmembrane transport
18	nucleolus
19	apoplast
20	cytosolic ribosome
21	peroxisome
22	microbody
23	proteasome complex
24	ribosome
25	endopeptidase complex
26	cytosolic small ribosomal subunit
27	endosome membrane
28	small ribosomal subunit
29	peptidase complex
30	endosome
31	SNARE complex
32	proteasome regulatory particle, base subcomplex
33	late endosome membrane
34	ribosomal subunit
35	perinuclear region of cytoplasm
36	transport vesicle
37	protein-containing complex binding
38	GTPase activity
39	translation elongation factor activity
40	pyrophosphate hydrolysis-driven proton transmembrane transporter activity
41	ion transmembrane transporter activity, phosphorylative mechanism

42	translation factor activity, RNA binding
43	proton-exporting ATPase activity, phosphorylative mechanism
44	ATPase-coupled cation transmembrane transporter activity
45	translation regulator activity, nucleic acid binding
46	amide binding
47	translation regulator activity
48	polyubiquitin modification-dependent protein binding
49	actin filament binding
50	ATPase-coupled ion transmembrane transporter activity
51	purine nucleoside binding
52	GTP binding
53	guanyl nucleotide binding
54	purine ribonucleoside binding
55	guanyl ribonucleotide binding
56	copper ion binding
57	nucleoside binding
58	ribonucleoside binding
59	sulfur compound binding
60	primary active transmembrane transporter activity
61	active ion transmembrane transporter activity
62	SNAP receptor activity

400 mM NaCl

1	response to cadmium ion
2	response to metal ion
3	cellular response to unfolded protein
4	response to unfolded protein
5	response to virus
6	cellular response to topologically incorrect protein
7	response to heat
8	protein folding
9	response to topologically incorrect protein
10	water transport
11	fluid transport
12	immune effector process
13	alpha-amino acid metabolic process
14	glutamine family amino acid biosynthetic process
15	protein refolding
16	cation transmembrane transport
17	monovalent inorganic cation transport
18	ion transmembrane transport
19	NADP metabolic process
20	L-phenylalanine catabolic process
21	erythrose 4-phosphate/phosphoenolpyruvate family amino acid catabolic process

22	'de novo' posttranslational protein folding
23	chaperone cofactor-dependent protein refolding
24	vitamin E biosynthetic process
25	vitamin E metabolic process
26	'de novo' protein folding
27	fat-soluble vitamin metabolic process
28	fat-soluble vitamin biosynthetic process
29	glycolytic process
30	ATP generation from ADP
31	purine nucleoside diphosphate metabolic process
32	purine ribonucleoside diphosphate metabolic process
33	ADP metabolic process
34	glutamine metabolic process
35	lignin biosynthetic process
36	cytosolic ribosome
37	ribosome
38	nucleolus
39	apoplast
40	cytosolic small ribosomal subunit
41	proteasome complex
42	ribosomal subunit
43	small ribosomal subunit
44	endopeptidase complex
45	ubiquitin-like protein ligase binding
46	protein tag
47	ubiquitin protein ligase binding
48	copper ion binding
49	misfolded protein binding
50	enzyme binding
51	protein folding chaperone
52	protein heterodimerization activity
53	water transmembrane transporter activity
54	water channel activity
55	structural molecule activity
56	heat shock protein binding
57	pyrophosphate hydrolysis-driven proton transmembrane transporter activity
58	phosphatidylinositol kinase activity
59	carbon-carbon lyase activity
60	unfolded protein binding
61	ubiquitin-like modifier activating enzyme activity
62	ATPase-coupled cation transmembrane transporter activity
63	oxidoreductase activity, acting on the aldehyde or oxo group of donors
64	aldehyde-lyase activity

65	ATPase activity, coupled to transmembrane movement of ions, rotational mechanism
66	proton-transporting ATPase activity, rotational mechanism
