# Supplementary material for: Comparative Ubiquitination Proteomics Revealed the Salt Tolerance Mechanism in Sugar Beet Monomeric Additional Line M14
Source: Int J Mol Sci. 2022 Dec 17;23(24):16088. doi: 10.3390/ijms232416088 (PMC9782053; doi:10.3390/ijms232416088)
Supplement: Supplementary file 1 [file ijms-23-16088-s001.zip › Table S7-List of KEGG enrichment entries.pdf]

Supplementary Table S7 List of KEGG enrichment entries

200 mM NaCl

1	Proteasome
2	Carbon fixation in photosynthetic organisms
3	Arachidonic acid metabolism
4	Spliceosome
5	Fructose and mannose metabolism
6	Glycolysis / Gluconeogenesis
7	Inositol phosphate metabolism
8	Phenylpropanoid biosynthesis
9	Phagosome
10	Biosynthesis of amino acids
11	Nitrogen metabolism
12	Fatty acid degradation
13	SNARE interactions in vesicular transport
14	Pyruvate metabolism
15	Biosynthesis of nucleotide sugars
16	Glutathione metabolism
17	Nucleocytoplasmic transport
18	Oxidative phosphorylation
19	Vitamin B6 metabolism
20	Pentose phosphate pathway
21	Citrate cycle (TCA cycle)
22	Tryptophan metabolism
23	Ribosome
24	Selenocompound metabolism
25	mRNA surveillance pathway
26	Cysteine and methionine metabolism
27	Amino sugar and nucleotide sugar metabolism
28	Phosphatidylinositol signaling system
29	Glyoxylate and dicarboxylate metabolism
30	Flavonoid biosynthesis
31	Ether lipid metabolism
32	Carbon metabolism
33	Purine metabolism
34	Peroxisome
35	Protein processing in endoplasmic reticulum
36	Endocytosis
37	Phenylalanine metabolism
38	Ribosome biogenesis in eukaryotes
39	Arginine biosynthesis
40	Glycerophospholipid metabolism
41	Cutin, suberine and wax biosynthesis

42	Ubiquinone and other terpenoid-quinone biosynthesis
43	Tyrosine metabolism
44	Sulfur metabolism
45	Base excision repair
46	Fatty acid biosynthesis
47	alpha-Linolenic acid metabolism
48	N-Glycan biosynthesis
49	DNA replication
50	Alanine, aspartate and glutamate metabolism
51	Ascorbate and aldarate metabolism
52	Biosynthesis of various plant secondary metabolites
53	Homologous recombination
54	Glycerolipid metabolism
55	Nucleotide excision repair
56	Fatty acid metabolism
57	Ubiquitin mediated proteolysis
58	2-Oxocarboxylic acid metabolism
59	Plant-pathogen interaction
60	Biosynthesis of cofactors
61	Starch and sucrose metabolism
62	Plant hormone signal transduction

400 mM NaCl

1	Biosynthesis of amino acids
2	Carbon fixation in photosynthetic organisms
3	Carbon metabolism
4	Nitrogen metabolism
5	Pentose phosphate pathway
6	Glutathione metabolism
7	Ubiquitin mediated proteolysis
8	Protein processing in endoplasmic reticulum
9	Proteasome
10	Glycolysis / Gluconeogenesis
11	Arginine biosynthesis
12	Ribosome
13	Alanine, aspartate and glutamate metabolism
14	Ascorbate and aldarate metabolism
15	Cysteine and methionine metabolism
16	Tryptophan metabolism
17	Arachidonic acid metabolism
18	Glyoxylate and dicarboxylate metabolism
19	Phagosome
20	Endocytosis
21	Oxidative phosphorylation

22	Pyruvate metabolism
23	Phenylalanine metabolism
24	Pentose and glucuronate interconversions
25	Spliceosome
26	Ubiquinone and other terpenoid-quinone biosynthesis
27	Circadian rhythm - plant
28	Tyrosine metabolism
29	Autophagy - other
30	alpha-Linolenic acid metabolism
31	RNA polymerase
32	Arginine and proline metabolism
33	Biosynthesis of cofactors
34	Citrate cycle (TCA cycle)
35	Fructose and mannose metabolism
36	Biosynthesis of various plant secondary metabolites
37	Homologous recombination
38	Nucleotide excision repair
39	Glycine, serine and threonine metabolism
40	2-Oxocarboxylic acid metabolism
41	Phosphatidylinositol signaling system
42	Peroxisome
43	Glycerophospholipid metabolism
44	Biosynthesis of nucleotide sugars
45	Nucleocytoplasmic transport
46	RNA degradation
47	Phenylpropanoid biosynthesis
48	Amino sugar and nucleotide sugar metabolism
49	Plant hormone signal transduction
