# Supplementary material for: Comparative Ubiquitination Proteomics Revealed the Salt Tolerance Mechanism in Sugar Beet Monomeric Additional Line M14
Source: Int J Mol Sci. 2022 Dec 17;23(24):16088. doi: 10.3390/ijms232416088 (PMC9782053; doi:10.3390/ijms232416088)
Supplement: Supplementary file 1 [file ijms-23-16088-s001.zip › Table S8-Sugar beet M14 line root proteins with both phosphorylations and ubiquitination.pdf]

Supplementary Table S8 Sugar beet M14 line root proteins with both phosphorylations and ubiquitination

Accessioin [a]	Tair [b]	Uniprot [c]	Gene name[d]	Protein name[e]
731369885	AT4G05200	Q9M0X5	CRK25	Cysteine-rich receptor-like protein kinase 25
731370431	AT4G03000	Q0WPJ7	RF298	Putative E3 ubiquitin-protein ligase RF298
731373041	AT4G05090	Q9M0Y6	At4g05090	Putative PAP-specific phosphatase
731374019	AT4G02570	Q94AH6	CUL1	Cullin-1
731374410	AT3G59350	B9DFG5	PTI13	PTI1-like tyrosine-protein kinase 3
731377617	AT4G23160	O65468	CRK8	Cysteine-rich receptor-like protein kinase 8
731320933	AT1G61870	Q8LE47	PPR336	Pentatricopeptide repeat-containing protein At1g61870
731321657	AT1G68200	Q9C9F5	At1g68200	Zinc finger CCCH domain-containing protein 15
731323540	AT5G30510	Q93VC7	RPS1	30S ribosomal protein S1
731323749	AT5G27990	Q8L9R4	At5g27990	At5g27990

731324314	AT5G27550	F4K4C5	KIN14S	Kinesin-like protein KIN-14S
731324480	AT5G38640	Q9FFV8	MBB18.19	At5g38640
731312213	AT5G50780	F4KAF2	MORC4	Protein MICRORCHIDIA 4
731325154	AT1G26480	Q9C5W6	GRF12	14-3-3-like protein GF14 iota
731327767	AT5G39775	A0A1P8BH06	At5g39775	Gag-Pol polyprotein/retrotransposon
731327882	AT3G45980	Q0WT91	At3g45980	Histone H2B
731312437	AT5G49160	A0A1P8BCY7	MET1	DNA (cytosine-5)-methyltransferase
731328696	AT5G24750	F4KII1	At5g24750	Glycosyltransferase
731329877	AT5G59970	P59259	At1g07660	Histone H4
731310789	AT3G02520	Q96300	GRF7	14-3-3-like protein GF14 nu
731330815	AT3G43920	Q9LXW7	DCL3	Endoribonuclease Dicer homolog 3
731333780	AT5G16350	A0A1P8BHJ4	MQK4.7	O-acyltransferase (WSD1-like) family protein
731333855	AT4G00430	F4JHB6	PIP1:4	Plasma membrane intrinsic protein 14

731334547	AT2G43850	F4IS56	ILK1	Integrin-linked protein kinase 1
731335064	AT4G01570	Q8VZE4	At4g01570	Pentatricopeptide repeat-containing protein At4g01570
731335068	AT4G01100	F4JHS4	ADNT1	Adenine nucleotide transporter 1
731310858	AT1G06970	Q9LMJ1	CHX14	Cation/H^+ antiporter 14
731337443	AT5G14080	Q9FMU2	At5g14080	Pentatricopeptide repeat-containing protein At5g14080
731337807	AT5G65780	Q9FYA6	BCAT5	Branched-chain-amino-acid aminotransferase 5
731339186	AT4G00980	Q8GXX5	A_TM018A10.1	At4g00980
731339333	AT3G13080	Q9LK64	ABCC3	ABC transporter C family member 3
731339519	AT2G26910	O81016	ABCG32	ABC transporter G family member 32
731340192	AT4G19210	Q8LPJ4	ABCE2	ABC transporter E family member 2
731313765	AT3G54300	Q9M376	VAMP727	Vesicle-associated membrane protein 727
731343378	AT3G62880	Q9LZH8	OEP164	Outer envelope pore protein 16-4

731343400	AT3G04400	P49690	RPL23A	60S ribosomal protein L23
731347368	AT3G02890	A0A178VJR1	At3g02890	Uncharacterized protein
731348334	AT5G53460	Q9LV03	GLT1	Glutamate synthase 1
731348961	AT5G16340	Q9FFE9	AAE6	Probable acyl-activating enzyme 6
731349098	AT1G13440	Q56WJ4	At1g13440	Putative glyceraldehyde-3-phosphate dehydrogenase
731349965	AT1G24360	P33207	At1g24360	3-oxoacyl-[acyl-carrier-protein] reductase
731351524	AT3G55440	P48491	CTIMC	Triosephosphate isomerase
731353170	AT1G18640	O82796	PSP	Phosphoserine phosphatase
731353294	AT4G34200	O49485	PGDH1	D-3-phosphoglycerate dehydrogenase 1
731353543	AT1G04410	B9DHX4	At1g04410	Malate dehydrogenase
731354228	AT2G21660	C0Z2N6	At2g21660	AT2G21660 protein
731355163	AT2G19710	O82206	At2g19710	Uncharacterized protein At2g19710

731358209	AT2G06105	A0A1P8B0V7	At2g06105	Aspartyl protease gag-polyprotein
731358562	AT1G21440	Q501F7	At1g21440	At1g21440
731359337	AT3G10300	F4J2M3	At3g10300	Calcium-binding EF-hand family protein
731359729	AT4G13230	Q8LFD5	At4g13230	Uncharacterized protein At4g13230
731360225	AT1G21750	F4HZN9	PDIL1-1	Protein disulfide-isomerase
731360885	AT1G21680	Q9XI10	At1g21680	DPP6 N-terminal domain-like protein
1108779495	AT3G10080	Q9SR72	At3g10080	Germin-like protein subfamily 3 member 2
731361122	AT1G22660	F4I2Y2	At1g22660	Polynucleotide adenylyltransferase family protein
731362572	AT5G66390	Q9FJZ9	PER72	Peroxidase 72
731362650	AT1G77120	P06525	ADH1	Alcohol dehydrogenase class-P
731364835	AT3G11840	Q570W6	At3g11840	Uncharacterized protein At3g11840
731366438	AT1G30580	Q9SA73	YchF1	Obg-like ATPase 1
731311222	AT2G17060	F4IMF2	At2g17060	ADP-ribosyl cyclase/cyclic ADP-ribose hydrolase

731366903	AT3G28750	A0A654FBJ9	AN1_LOCUS14381	Uncharacterized protein
731367408	AT4G03500	Q9ZT78	At4g03500	Ankyrin repeat family protein
731367897	AT3G15570	Q8LFK3	At3g15570	Non-phototropic hypocotyl protein
1108824977	AT1G55490	A0A178W724	AXX17_At1g50100	hypothetical protein
1108865430	AT1G75040	A0A178WNL8	AXX17_At1g69430	PR5
1108872178	AT4G19210	Q8LPJ4	ABCE2	ABC transporter E family member 2
1108892120	AT5G54850	Q9FFU5	MBG8.11	At5g54850
1108902428	AT1G07840	Q93ZQ9	At1g07840	AT1G07840 protein
1108931327	AT3G17152	Q9LSN2	At3g17152	Invertase inhibitor-like protein
1108938555	AT4G13510	A0A178V540	AXX17_At4g15360	Ammonium transporter
1108953901	AT1G43760	Q9MA67	F2J6.14	F2J6.14 protein
1108972321	AT1G64940	Q9XIQ2	CYP89A6	Cytochrome P450
731378086	AT5G14580	A0A5S9Y435	AT9943_LOCUS19138	Polyribonucleotide nucleotidyltransferase

731381505	AT4G33145	Q1G3K5	At4g33145	Uncharacterized protein
731311597	AT4G34320	Q9SYZ7	At4g34320	UPF0496 protein At4g34320
731320688	AT4G31580	A0A5S9XYC9	AN1_LOCUS19964	hypothetical protein

[a] Protein sequence number in the NCBI database. [b] *Arabidopsis* TAIR number. [c] Uniport protein number.
