# Supplementary material for: Comparative Ubiquitination Proteomics Revealed the Salt Tolerance Mechanism in Sugar Beet Monomeric Additional Line M14
Source: Int J Mol Sci. 2022 Dec 17;23(24):16088. doi: 10.3390/ijms232416088 (PMC9782053; doi:10.3390/ijms232416088)
Supplement: Supplementary file 1 [file ijms-23-16088-s001.zip › Table S9-qRTPCR-primers.pdf]

Supplementary Table S9 Primer sequences required for the assay

Primer name	Primer sequences
E1-1 F	5'-CAGGATGAACCCAGTGCAGT-3'
E1-1 R	5'-TGCAGTGGACGTTGCAATTG-3'
E1-2 F	5'-TTGTGACGGATGAGAAGGCC-3'
E1-2 R	5'-AGTTCCTCGCCCTCATGTTG-3'
E2-2 F	5'-GTCCTGATGACACCCCTTGG-3'
E2-2 R	5'-ACATCCGTGCAGCTTCTGAA-3'
E2-27 F	5'-CCTTTCGAGCCACCCTTGAT-3'
E2-27 R	5'-TTGAGCAACGACAGCATCCT-3'
E3-BRE1 F	5'-CGGCGTCACGTAAGCTCTAT-3'
E3-BRE1 R	5'-AGCTTCTGGCAGAGCTTCTG-3'
E3-CHIP F	5'-CATATTCCGCGACCCTGTCA-3'
E3-CHIP R	5'-GAGTAAGTGGTTCCCGGGTG-3'
E3-UPL5 F	5'-CGGTCGTGATGGGTCTGAAA-3'
E3-UPL5 R	5'-TTTCAAAGGCCCAATGCTGC-3'
18S-F	5'-CCCCAATGGATCCTCGTTA-3'
18S-R	5'-TGACGGAGAATTAGGGTTCG-3'
